# Women in Healthy Transition (KISO) Survey: a cohort of 153,800 women aged 45–59 years living in Denmark

**DOI:** 10.1007/s10654-025-01291-0

**Published:** 2025-08-27

**Authors:** Sigrid Normann Biener, Terese Sara Høj Jørgensen, Maria Hybholt

**Affiliations:** 1 Department of Public Health, University of Copenhagen, Copenhagen, Denmark; 2 Department of Nutrition, Exercise and Sports, University of Copenhagen, Copenhagen, Denmark; 3 Center for Clinical Research and Prevention, Copenhagen University Hospital, Bispebjerg, Frederiksberg, Denmark

**Keywords:** Menopause, Menopausal symptoms, Women’s health, Cohort profile

## Abstract

The nationwide Women in Healthy Transition (KISO) Survey Cohort is a population-based longitudinal prospective cohort study established to explore the significant data gap on women’s symptoms through different stages of menopause in a Northern European context. The KISO Survey Cohort was set up to represent women aged 45–59 years living in Denmark. In total 575,863 women were invited to participate in the study at baseline. Data were collected through digital questionnaires from June to December 2024 and included self-reported information on stages of menopause, menopausal symptoms, quality of life, physical activity, and work productivity loss using validated scales as well as information on various health, social and lifestyle factors. The follow-up of the KISO Survey Cohort will be conducted through digital questionnaires every three years over a 15-year period, inviting baseline respondents and eligible women aged 45–59 at follow-up. A total of 153,800 women completed the baseline questionnaire, yielding a 27% response rate. Among the participants, 8% were in premenopause, 24% in perimenopause, and 45% in postmenopause. Moreover, 13% had induced menopause and 10% were undergoing menopausal hormone therapy. The KISO Survey Cohort is the first large-scale longitudinal study on menopausal symptoms among women in Denmark. Data are coupled with the personal identification numbers (CPR) enabling opportunities to link data to national administrative registers. This ongoing study, thus, offers unique and extensive data, enabling future research to advance our understanding of menopause, how it affects women, and its long-term effects on women.

**Supplementary Information:**

The online version contains supplementary material available at 10.1007/s10654-025-01291-0.

## Introduction

Menopause is a natural phase in the biological aging process of women marking the end of the reproductive years. It is generally divided into three stages involving premenopause, perimenopause and postmenopause which is classified according to hormonal status and menstrual bleeding patterns. Hormonal changes affect women differently and vary across the stages of menopause [1]. During menopause, some women experience prolonged periods of physical and psychological symptoms that hinder their everyday life [2].

Globally, there is considerable variation in the prevalence and types of menopausal symptoms reported by women across cultures and ethnic groups [3–6]. For instance, studies indicate that Black women experience a higher prevalence of hot flushes compared to White, Hispanic, and Asian women, while White women report more psychosomatic symptoms than Black, Hispanic, and Asian women [3, 7].

Overall, a meta-analysis (27 studies, n = 191,830) and a systematic review (55 studies, n = 76,817) report variations in the prevalence of hot flushes ranging from 18% to 98%, sleep disturbances from 20% to 86%, and depressive symptoms from 6% to 87% [7, 8]. This significant discrepancy in findings underscores the need for longitudinal cohort studies to explore how women experience symptoms throughout menopause with information spanning from premenopause through perimenopause to postmenopause. To our knowledge, a population-based longitudinal prospective cohort study on menopausal symptoms has not previously been collected in a Northern European context. Therefore, we have developed and established the nationwide Women in Healthy Transition (KISO) Survey Cohort with plan follow-up every three years for 15 years including all previous respondents as well as eligible women aged 45–59 years at each follow-up.

This Cohort Profile presents the study design, follow-up procedure, and baseline characteristics as a foundational resource for future research. The main purpose of the study is to gain a better understanding of menopause, how it affects women, and its long-term effects on women living in Denmark and thereby fill an important data gap by exploring women’s symptoms during the different stages of menopause.

## Methods

### Study design

The KISO Survey Cohort was designed as a prospective cohort study targeting women living in Denmark to create a large-scale cohort with planned follow-up questionnaires every three years. Data are collected through digital self-administered questionnaires in the Danish survey platform SurveyXact linked to the personal identification number (the Central Personal Registration (CPR) number). This provides an opportunity to link data to national administrative registers with information about health and sociodemographic factors.

### Study population and data collection

The KISO Survey Cohort was set up to represent women aged 45–59 years living in Denmark. The target population was identified in the CPR register based on the following inclusion criteria at the time of extraction: female civil registration number, aged 45–59 years, alive and living in Denmark (excluding Greenland), no name and address protection, and not being legally incapacitated. This yielded a total of 575,863 women^1^ in the target population at baseline. Research Services under the Danish Health Data Authority prepared and delivered the contact information (CPR numbers and names) on 21 June 2024.

Between 24 and 26 June 2024, invitation letters with a digital link to the KISO Survey Cohort baseline questionnaire were sent to the target population via Digital Post.^2^ Reminder letters were sent between 22 and 24 October 2024 to women who had not responded to the first invitation and to women who had partially responded. The questionnaire closed on 9 December 2024.

**Fig. 1:**
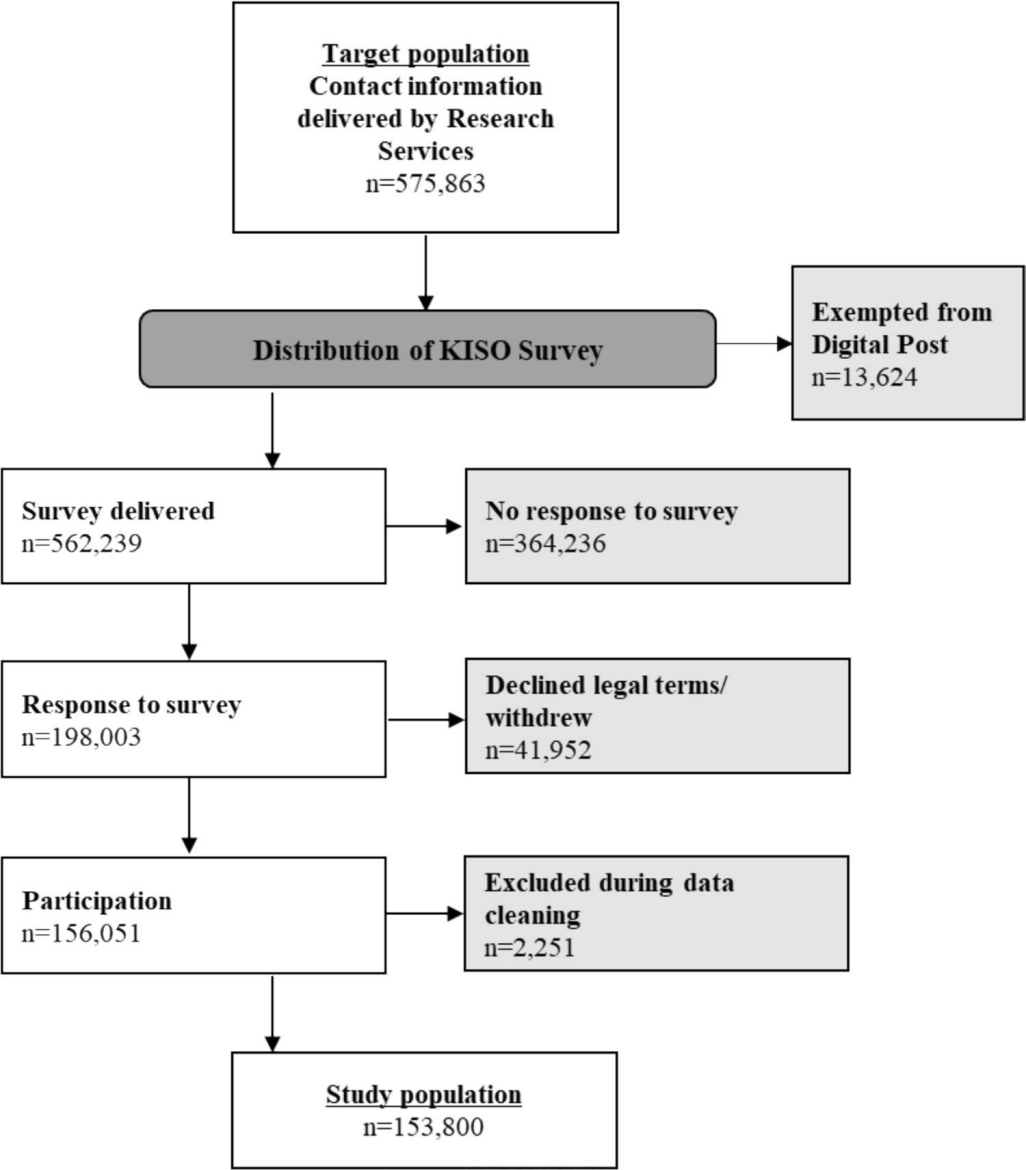
Flow chart of the KISO Survey Cohort baseline 2024

Figure 1 shows the flow chart of the final KISO Survey Cohort 2024. Among the 575,863 women initially extracted for the survey, 13,624 were excluded because they were registered as exempt from Digital Post and therefore did not receive the invitation. In total, 364,236 women did not respond to the survey and 41,161 either did not approve the lawful basis of research or withdrew from the survey via mail notification. During data cleaning, 2,251 women were excluded due to missing response to menopause-specific questions (n = 2,198) or for providing incorrect day, month, and year of birth, indicating unreliable responses (n = 53).

This left a total study population of 153,800 women at baseline, including both complete and partially complete responses (n = 139,178 and n = 14,622, respectively) corresponding to a participation rate of 27%.

### Baseline characteristics of KISO Survey Cohort

A total of 153,800 women were included in the baseline study. Table 1 presents baseline characteristics of the study population included in the KISO Survey Cohort juxtaposed with characteristics of all women aged 45–59 years living in Denmark on 1 January 2024 retrieved from Statistics Denmark (background population). There is a difference in the proportion of women in the three age groups, with a lower proportion of women aged 45–49 years (27% vs. 32%) participating in the KISO Survey Cohort compared with the background population, and a slightly higher proportion of women aged 50–54 years (36% vs. 33%) and 55–60 years (37% vs. 35%), respectively, among the KISO participants compared with the background population. In terms of municipality of residency, we found a similar proportion of women living in urban, suburban, and rural areas among women in the KISO Survey Cohort and in the background population. A higher proportion of women with Danish origin (93% vs. 85%), higher education (65% vs. 45%), and in employment (85% vs. 81%) participated in the study compared with the background population. The proportions of cohabitation status and parity were similar among women in the KISO Survey Cohort and the background population.

**Table 1:** Baseline characteristics of the study population of KISO Survey Cohort and background population data from Statistics Denmark

	KISO Survey Cohort n (%)		Statistics Denmark n (%)	
Total	153,800	(100%)	592,535	(100%)
Age				
45–49 years	42,249	(27%)	189,003	(32%)
50–54 years	55,323	(36%)	195,716	(33%)
55–60 years	56,228	(37%)	207,816	(35%)
*Missing*	*0*			
Municipality group				
Urban	60,184	(39%)	227,109	(38%)
Suburban	64,502	(42%)	245,967	(42%)
Rural	29,076	(19%)	119,459	(20%)
*Missing*	*38*			
Ethnicity				
Danish origin	142,766	(93%)	502,677	(85%)
Immigrant	10,122	(7%)	86,457	(15%)
Descendant	700	(< 1%)	3,401	(1%)
*Missing*	*212*			
Education^a^				
Less than high school	5,055	(3%)	82,176	(14%)
High school or vocational training	44,398	(29%)	240,239	(40%)
Higher education	99,678	(65%)	264,910	(45%)
Other/don’t know	4,669	(3%)	6,781	(1%)
*Missing*	*0*			
Occupation^b^				
Employed	131,240	(85%)	478,237	(81%)
Unemployed	2,574	(2%)	8,894	(2%)
Outside the labor force	11,5332	(7%)	105,641	(18%)
Other/don’t know	8,454	(5%)		
*Missing*	*0*			
Parity				
Multiparity	135,806	(88%)	521,887	(88%)
Nulliparity	17,973	(12%)	70,648	(12%)
*Missing*	*21*			
Cohabitation				
Living with others	132,801	(86%)	500,073	(84%)
Living alone	20,978	(14%)	92,462	(16%)
*Missing*	*21*			

*Source* Data from Statistics Denmark on women aged 45–59 years, 01.01.2024 (n = 592,535)

^a^Data from Statistics Denmark on women aged 45–59 years, 01.01 2023 (n = 594,106)

^b^Data from Statistics Denmark on women aged 45–59 years, 30.11.2023 (n = 592,772)

*Note* Not all percentages sum to 100% because of rounding

### Development and testing of the questionnaire

The baseline questionnaire comprises one international standard system and four validated scales to measure stages of menopause [1], menopausal symptoms [10], quality of life (QOL) [11], physical activity (PA) [12], and work productivity loss [13], respectively.

Validated scales were selected through a comprehensive literature review to identify the most relevant and comparative measures for the target group and field of study. Scales not available in Danish were translated using a forward–backward translation method [14]. Additionally, we developed closed-ended items to assess various health, social, and lifestyle conditions. The questionnaire underwent an extensive testing process, with a pilot survey tested and revised several times. We conducted three main types of testing: pre-testing with survey researchers (n = 2) and pilot testing (n = 14) and cognitive interviews (n = 8) with the target group. Validity was assessed for content validity, construct validity, and criterion validity [14].

### Measurement indicators

*Stages of menopause*: Menopausal status was measured following the Stages of Reproductive Aging Workshop (STRAW) + 10 criteria, based on menstrual bleeding patterns. The STRAW + 10 staging system is considered the golden standard for defining reproductive aging through menopause. We used specification of the menstrual cycle criteria to measure stages of menopause as follows: regular menstrual cycle indicated the premenopause (reproductive) stage, changes in menstrual cycle where the woman had had menstrual bleeding within the last year indicated perimenopause, and one year or more since the final menstrual period (FMP) was defined as postmenopause. Postmenopause was stratified into three subcategories: early postmenopause (1–2 years), late early postmenopause (> 2–8 years), and late postmenopause (> 8 years),^3^ based on the STRAW + 10 criteria [1]. In addition, women with induced menopause due to surgical or medical treatment and women with current use of menopausal hormone therapy (MHT) were stratified into separate groups. Women who fell into multiple categories were classified based on the following priority: induced menopause, MHT usage, and stage of menopause. Hormonal contraceptives may affect women’s natural menstrual cycle and thereby lead to misclassification of menopausal stage [15]. Thus, sensitivity analyses were conducted where women using hormonal contraception were included as a separate category.

*Menopausal symptoms.* Menopausal symptoms were measured with The Menopause Rating Scale (MRS) scale comprising 11 items divided into three domains of symptoms, including psychological symptoms, somato-vegetative symptoms, and urogenital symptoms [10]. The scoring points of the MRS scale indicated severity of symptoms perceived by the women ranging from 0 (“no complaints”) to 4 (“extremely severe”). The composite scores for each domain of symptoms were based on adding up the scores of each item of the respective domain, while the total score was the sum of the domains scores. The total score and domain scores were evaluated in weighted categories of severity rating from “no, little” over “mild” and “moderate” to “severe”. [10]. In addition to the MRS scale, based on the results from the pilot test and other menopause-related scales [16–18], we added the following five additional symptoms to the questionnaire: night sweats, skin changes, headache, rage, and dizziness using the same scoring points as in the MRS scale.

*Quality of life:* QOL was measured with the Utian Quality of Life (UQOL) scale consisting of 23 items designed to measure quality of life of women in peri- and postmenopause [11]. The scale incorporates sense of well-being reflecting QOL specific to women in menopause that extend beyond menopausal symptoms divided into four QOL-domains: occupational QOL, health QOL, emotional QOL, and sexual QOL. The scoring for each item included five-point Likert-type scale rating from 1 (“not true of me”) to 5 (“very true of me”) and the total score was measured by adding the scores of each item [11].

*Physical activity*: PA was measured using the International Physical Activity Questionnaire (IPAQ) long form self-administered format [12]. The IPAQ long form consist of detailed questions about walking, moderate-intensity, and vigorous-intensity activities in each of the four domains, including work-related PA, transport-related PA, domestic and gardening activities, and leisure time-based PA [12]. All activities were converted to Metabolic Equivalent Task (MET) scores, summed to obtain the total MET-minutes/week, and then categorized into three levels of PA: “low”, “moderate”, and “high” [19].

*Work productivity loss*: Menopause-related work productivity loss was measured using the Work Productivity and Activity Impairment (WPAI): Menopause questionnaire comprising six items. The WPAI: Menopause created a patient-reported quantitative assessment of the amount of absenteeism, presenteeism, and daily activity impairment attributable to menopausal symptoms [13, 20].

*Health, social, and lifestyle conditions:* Information on other health, social, and lifestyle conditions were measured as closed-ended questions. A comprehensive overview of topics and domains in the KISO Survey Cohort is available in Table 2.

**Table 2:** Overview of data in the baseline questionnaire of the KISO Survey Cohort

Topics	Domains
General information	
Demographics	Age, ethnicity, municipality of residence
Social status	Education, occupation
Social relations	Partner, cohabitation, social support
Lifestyle	
Physical activity	IPAQ—long form
Alcohol habits	Alcohol consumption in days and units per week
Smoking	Smoking status
Sleep	Sleep pattern
Body and health	
Body weight	Hight, weight, weight perception, weight loss desire
Reproduction	Contraceptive history, abortion history, parity
Menopause specifics	
Stages of menopause	Based on STRAW + 10, induced menopause (surgical or medical), MHT usage, contraceptive usage, pregnancy/lactation
Attitude	Attitude towards menopause
Support	Talk with medical doctor/other HCP, other support
Treatment	MHT use, non-hormone prescription drugs, supplements, alternative therapy
Symptoms and well-being	
Menopausal symptoms	MRS 11-items, 5 additional items (night sweats, skin changes, headache, rage, dizziness)
Quality of life	UQOL Scale 23-items
Work and activity productivity loss	WPAI: menopause 6-items

*Abbreviations* IPAQ, international physical activity questionnaire; STRAW, Stages of Reproductive Aging Workshop; MHT, menopausal hormone therapy; HCP, health care professional; MRS, Menopause Rating Scale; UQOL, Utian Quality of Life; WPAI, work productivity and activity impairment

### Follow-up procedures and data linkage

The follow-up of the KISO Survey Cohort will include repeated data collection with digital questionnaires every three years for a 15-year period, at least yielding five future waves of data collection with the first wave scheduled in 2027. Eligibility for follow-up includes all women living in Denmark aged 45–59 years at the time of follow-up, as well as all women who completed the baseline questionnaire, regardless of their current age. Women who actively declined participation at the time of the baseline invitation will not be invited to participate in follow-up surveys. This approach ensures continuity in longitudinal data collection while also allowing for the inclusion of new participants within the target age range. We will use register-based information about all invited individuals (respondents and non-respondents) to develop inverse probability weights that can be used to mitigate possible selection bias from non-respondents who may be a selective group.

Follow-up measures will build upon the baseline questionnaire, allowing us to follow the progression of menopausal status, symptoms, QOL, and work productivity loss in relation to health, social, and lifestyle factors over time. The questionnaire will be continuously developed, with opportunities for refinement and expansion as new research priorities emerge.

At present, the data collected in the KISO Survey Cohort is linked with the CPR making it possible for future research projects to link the KISO Survey Cohort data to relevant nationwide registers including a wide range of social and health-related information. Future studies will for example explore how stages of menopause and menopausal symptoms are associated with a range of social outcomes, such as employment status and income, as well as health outcomes, including specific mental and somatic illness and patterns of healthcare utilization. These studies will be based on comprehensive data integration between KISO Survey Cohort and national administrative social and health data sources. In addition, qualitative studies will be conducted to capture the complexity and the nuanced experiences of menopause. These studies aim to provide an in-depth understanding of how menopause is perceived, managed, and experienced in everyday life, thereby complementing the quantitative findings with rich, contextual insights.

## Key findings

### Timing of the natural menopause

A Current Status analysis was performed on the baseline data from KISO Survey among women classified in premenopause, perimenopause, and postmenopause. Based on women’s age and whether they were in postmenopause or not, we estimated the median age at natural menopause to be 52.0 years. Additionally, a Kaplan–Meier survival analysis was applied on the same population, based on age and the retrospective information on the timing of FMP, which yielded a slightly lower median age at natural menopause of 51.4 years. The Current Status model, which does not incorporate retrospective information on the FMP, may offer greater reliability in cross-sectional data [21]. Both ages are consistent with previous findings on age at natural menopause among Caucasian women in industrialized countries (50–52 years) [22–25].

### Stages of menopause

In the study population, 11,747 women (8%) were classified as being in premenopause, 36,298 women (24%) being in perimenopause, and 67,165 women (45%) being in postmenopause. The latter group included 8,404 women in the early postmenopause (1–2 years), 36,539 women in late early postmenopause (> 2–8 years), and 22,222 women in late postmenopause (> 8 years). Furthermore, 20,367 women (13%) reported that they had experienced induced menopause. While 15,227 women (10%) were currently using MHT without having experienced induced menopause.

**Fig. 2:**
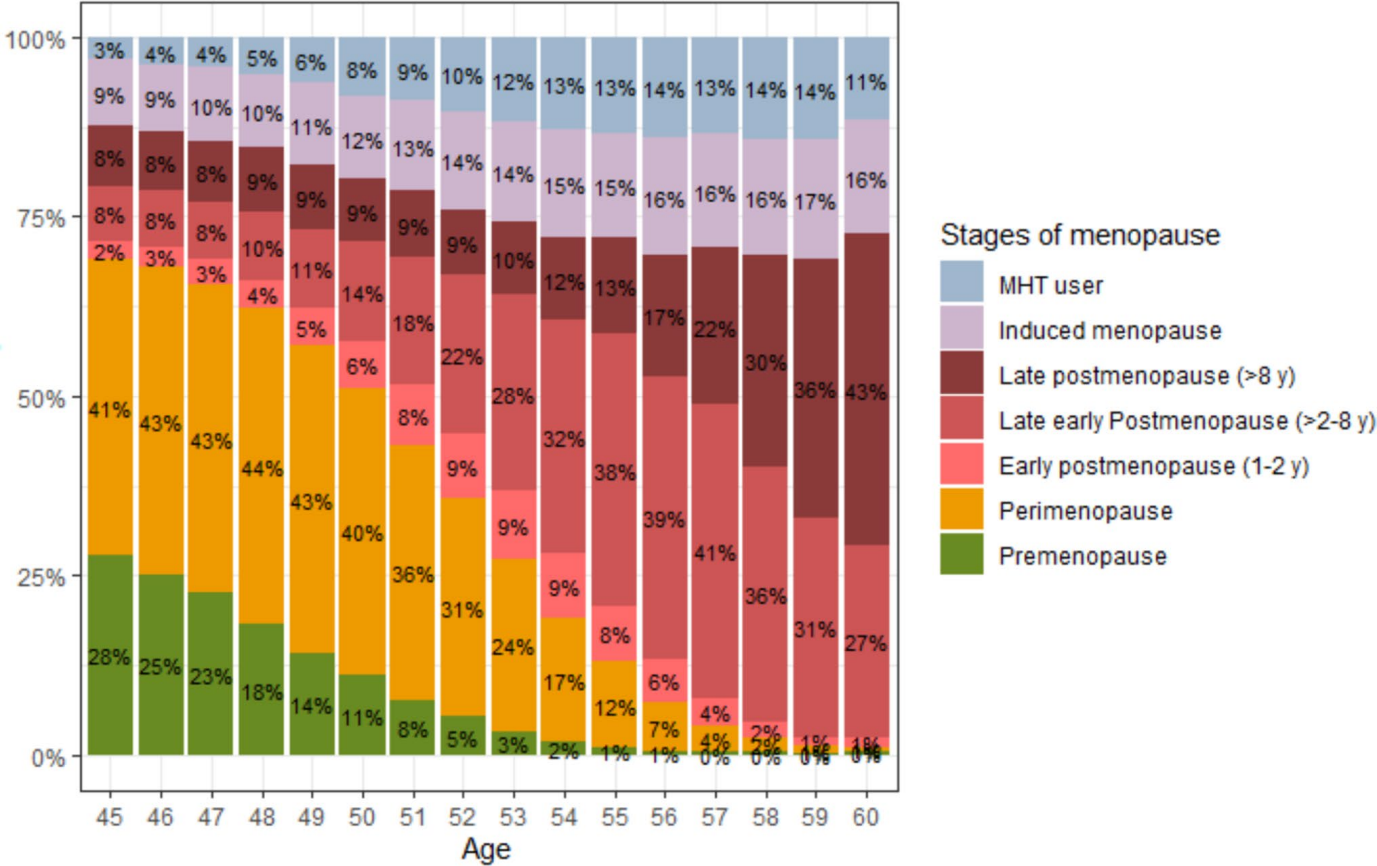
Stages of menopause at age 45–60 years. *Sample size:* n = 150,804. *Note:* The age range includes up to 60 years because a minor proportion of respondents answered the questionnaire after turning 60. *Abbreviations:* MHT, menopausal hormone therapy; y, years

In Fig. 2, we found that the stages of menopause naturally progressed with aging [22]. Before age 52, the proportion of women in pre- and perimenopause was greater than the proportion of women in postmenopause. However, after age 52, the proportion of women in postmenopause was greater than the proportion of women in pre- and perimenopause. The proportion of women with an induced menopause and MHT usage increased slightly with age.

Sensitivity analyses on stages of menopause at age 45–60 years dividing women using hormonal contraception into a separate category are available in the supplementary information (see Fig. S1a in Supplementary material 1).

### Menopausal symptoms

**Fig. 3:**
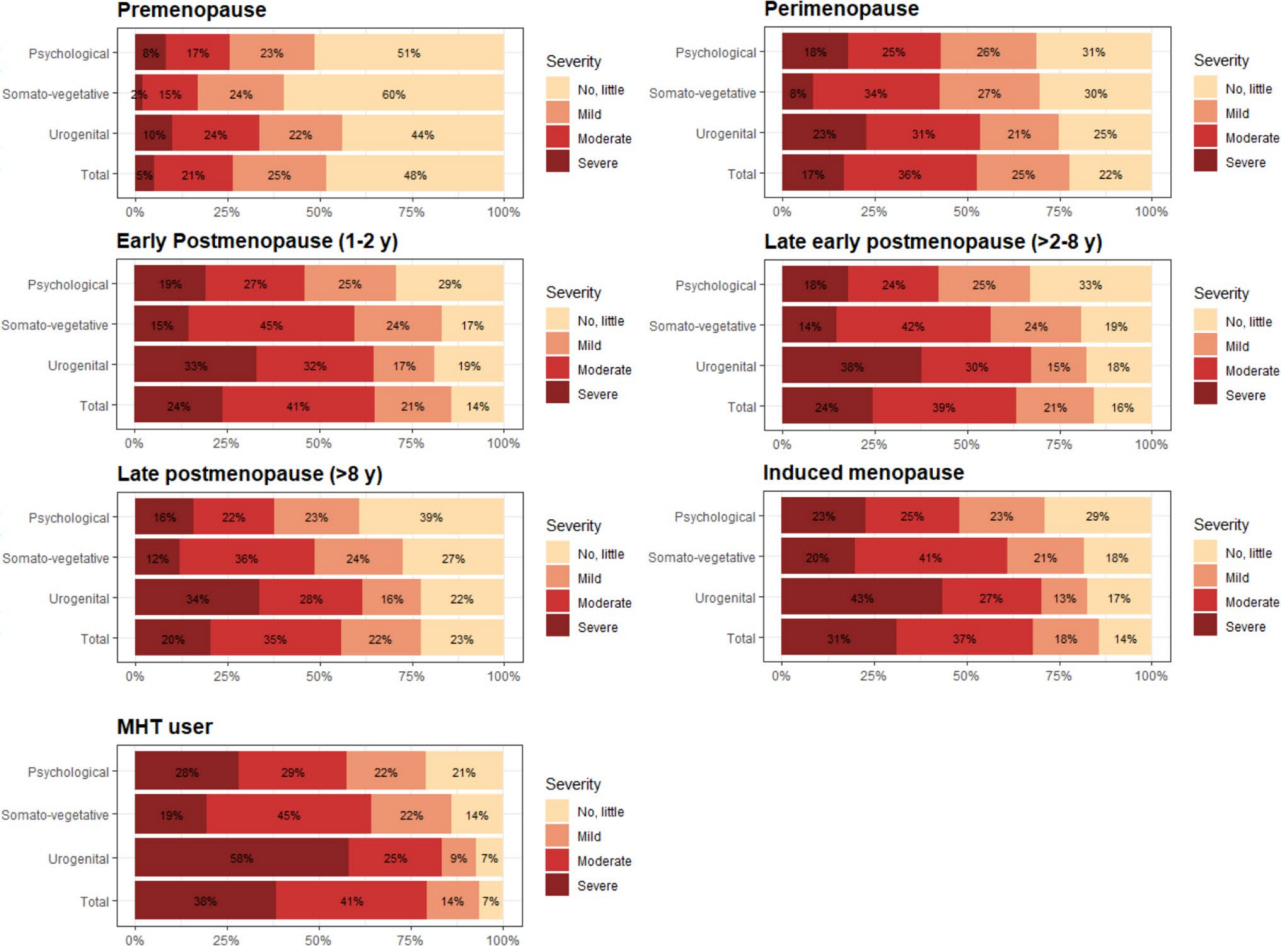
Prevalence and severity of symptom domains on stages of menopause. *Sample size*: premenopause (n = 11,659), perimenopause (n = 36,124*), early postmenopause (n = 8,314), late early postmenopause (n = 36,258*), late postmenopause (n = 21,932*), induced menopause (n = 20,102*), MHT user (n = 15,062*). *n varies by ≤ 5 across the 4 sub-analyses due to missing values. *Note*: Based on MRS 11-item symptoms. “Severe” and “extremely severe” were combined into “severe”. *Abbreviations*: MHT, menopausal hormone therapy; MRS, Menopause Rating Scale; y, years

Data on menopausal symptoms showed variance in prevalence and severity across stages of menopause. In Fig. 3, we found that women in premenopause reported the lowest prevalence and severity of total symptoms, with 48% experiencing no or little menopausal symptoms. We observed a higher prevalence and severity of symptoms among women in the perimenopausal stage with 17% experiencing severe symptoms compared to 5% in the premenopausal stage. Women in early postmenopause (1–2 years) reported the highest prevalence of total symptoms, with 24% experiencing severe symptoms. Symptom prevalence and severity slightly decreased in the later postmenopausal stages, but did not return to premenopausal levels. This progression in symptoms is somewhat consistent with the few previous studies that differentiate symptoms based on stages of menopause. These studies also showed that symptoms peaked around perimenopause and (early) postmenopause [2–5, 26].

Women with induced menopause and women who use MHT in general reported to be more affected by severe symptoms compared to women across the stages of menopause. This corresponds with previous findings of women with induced menopause reporting increased symptom severity compared to women with natural menopause [27], and MHT users experiencing symptoms despite MHT’s known effectiveness in alleviating symptoms, likely due to selection factors associated with MHT use [3].

Urogenital symptoms (including sexual problems, bladder problems, and vaginal dryness) represented the most severe symptom domain across all stages of menopause. The proportion of women experiencing these symptoms peaked during late early postmenopause (> 2–8 years), while the proportion of women experiencing psychological symptoms (including depressive mood, irritability, anxiety, and exhaustion) and somato-vegetative symptoms (including hot flashes, heart discomfort, sleep problems, and joint/muscular discomfort) peaked in the early postmenopause (1–2 years).

In supplementary information, prevalence and severity of individual symptoms across stages of menopause are available (see Fig. S2a–g in Supplementary material 2).

Sensitivity analyses on prevalence and severity of symptom domains dividing women using hormonal contraception into a separate category are available in the supplementary information (see Fig. S1b in Supplementary material 1).

## Discussion

KISO Survey Cohort comprises a baseline study population on 153,800 women living in Denmark born 1964–1979, representing 27% of the Danish source population, with extensive self-reported information on stages of menopause, menopausal symptoms, QOL, work productivity loss, and health, social, and lifestyle conditions. Key findings from the baseline data indicate that women in Denmark experience natural menopause at age 52.0. Furthermore, our findings indicate that 17–24% of women in perimenopause and postmenopause, respectively, experience severe symptoms compared to 5% of women in premenopause. Furthermore, 20% of the women in late postmenopause (> 8 years) report severe symptoms, indicating that significantly more women eight years or more after their FMP, when hormonal levels are supposedly stabilized, experience severe symptoms compared to those in premenopause.

A major strength of the KISO Survey Cohort is the large size of the baseline study population. This provides unique and extensive data on women’s self-reported stages of menopause and symptoms, along with various factors of relevance to menopause. These comprehensive data enable researchers to conduct studies that were previously unfeasible due to the lack of this specific type of data, significantly advancing our understanding of menopausal symptoms and their impact on women.

An additional strength is the standardized collection of data on stages of menopause, menopausal symptoms, QOL, PA, and work productivity loss using validated scales. By employing internationally standardized instruments, we enhance consistency and reliability as well as reduce measurement errors and increase the accuracy of data collection. Furthermore, we gain the ability to compare results with international research studies [28]. This contributes to the global research discourse on menopause.

Furthermore, a key strength of the KISO Survey Cohort lies in its longitudinal design, which enables the tracking of women over time. This is particularly valuable when studying transitional life phases, such as the menopausal transition. By following the same women from premenopause through perimenopause and into postmenopause, the cohort allows for a nuanced understanding of how biological, psychological, and social changes unfold across this period. This design not only enhances the ability to identify temporal patterns but also provides a robust framework for examining individual trajectories in menopausal experiences.

Another major strength of our study is the linkage to CPR making it possible to link survey data to the Danish administrative registers. Linkage to register-based information supports a unique opportunity to conduct detailed sub-analyses, sensitivity analyses as well as dropout analyses, comparing respondents with non-respondents. It will further be possible to identify women who belong to other cohorts with information on other psychosocial, behavioral, and biological conditions.

This study also has some weaknesses that need to be considered. Despite the large size of the baseline study population, the response rate of 27% is a concern because the participants were skewed with respect to age and certain social factors when compared to background data from Statistics Denmark. In total, we found five percentage points higher proportion of women within the age groups 50–54 years and 55–60 years among the participants compared to the background population. This suggest a tendency that women who were expected to be in the midst of menopause were more likely to participate than women in premenopause. Furthermore, it is likely that women who experienced more and intense menopausal symptoms were more willing to participate, which may have led to an overestimation of menopausal symptoms identified in the study. In addition, women in the KISO Survey Cohort represented a socially selected group, with a higher proportion of women with Danish origin (93% vs. 85%), and women with higher education (65% vs. 45%) compared to the background population. This pattern may reflect greater engagement with health research among these groups, possibly due to higher health literacy or fewer language and cultural barriers. Such differences could also be associated with symptom reporting and treatment use, as health awareness and healthcare access may vary by education and ethnicity. Selection bias is a well-recognized challenge in survey-based research. However, by linking survey data to national registers, we can develop statistical weights to adjust for selective participation. This approach helps improve the representativeness of the data and strengthens the validity of the findings [29]. Moreover, the representativeness of the KISO Survey Cohort participation will be further examined through sensitivity analyses and non-response analyses [30].

The relatively low response rate may partly be explained by (i) the survey being only available through Digital Post, (ii) the survey being comprehensive and estimated to take 30 min to complete, (iii) we had to revise the legal terms to ensure GDPR compliance following the distribution of the invitation letters, which requited participants to accept the new lawful basis of research, (iv) the survey questions were only available in Danish, and (v) the survey targeted all women within the specified age group and thus a diverse group including women with cognitive and physical disabilities. In addition, due to economic resources, we only sent one digital reminder letter throughout the survey period. More digital reminder letters, physical reminder letters, and reminders by phone calls had been shown to raise the response rates in other Danish studies [31–33]. However, there are also ethical considerations when repeatedly approaching citizens that must be taken into account.

Although the STRAW + 10 criteria are considered the golden standard for measuring stages of menopause, we recognize some limitations with this method. Firstly, STRAW + 10 does not offer standardized questions to assess its criteria. Secondly, menstrual bleeding history can be influenced by factors unrelated to menopause, such as stress and eating disorders [34, 35], which could lead to potential misclassification. Measurement of endocrines such as follicle-stimulating hormone, antimüllerian hormone, and Inhibin-B, along with antral follicle count, supports the classification of stages of menopause according to the STRAW + 10 standard [1]. Since neither Danish administrative registers nor Danish biobanks systematically collect such biomarkers, their inclusion as supportive measures for questionnaire responses tied to specific time points was precluded. Thus, in line with other midlife and menopausal cohort studies, such as the Women’s Health Initiative and Study of Women’s Health Across the Nation, we classified menopausal status based on self-administered data [36, 37]. Furthermore, a longitudinal study in a Northern European population found that estradiol levels supported questionnaire-based definitions of stages of menopause derived from the STRAW criteria [38]. Nevertheless, the development of a validated questionnaire based on the STRAW + 10 framework remains highly relevant for future research in the field of menopause, as it would provide a standardized tool for assessing stages of menopause. In addition, we identify a risk of misclassification because FMP was retrospectively defined as one year of amenorrhea [1]. Thus, women who had experienced FMP within a year were classified as being in perimenopause following the STRAW + 10 standards despite the likelihood of actually being in postmenopause, introducing an independent nondifferential measurement error [39]. Another consideration is that retrospective self-reporting of menstrual bleeding history may increase the risk of measurement error due to memory failure. This was particularly relevant for participants whose FMP had occurred a longer time ago, as it may be more challenging for them to accurately remember and report the timing of their last period. Consequently, this could have resulted in misclassifications within postmenopausal subgroups, but it would not have led to systematic bias [40]. As previously described, our findings regarding the median age at natural menopause were consistent with earlier studies, which strengthens the validity of our results.

Finally, in the KISO Survey Cohort, it was not possible to distinguish whether the reported symptoms were due to menopause or other diseases. Therefore, there is a risk that the symptom profiles were disturbed by other disease-related symptoms. We expect this to have the same effect on all stages of menopause, thereby reducing the relative difference between them.

In summary, the KISO Survey Cohort represents a valuable research resource with the potential to generate important insights about menopause, how it affects women, and its long-term effects on women that were previously unattainable due to the absence of this particular kind of data. With its prospective cohort design and linkage to national administrative registries, the cohort is well-positioned to support a wide range of epidemiological investigations over time. Future waves of data will enhance the cohort’s utility, enabling researchers to explore temporal trends and long-term health outcomes related to menopause in the Danish population. Moreover, the KISO Survey Cohort will support future qualitative studies aimed at unfolding the complexity and nuance of menopausal experiences and thereby enriching the overall understanding of this life phase.

## Collaborations

The steering committee of the KISO Survey Cohort welcomes collaboration and the interest of national and international colleagues. For more information, please contact the KISO steering committee [kisosurvey@ku.dk].

## Supplementary Information

Below is the link to the electronic supplementary material.

Supplementary file1 (PDF 308 kb)

Supplementary file2 (PDF 355 kb)

## Footnotes

1 In the KISO Survey Cohort, we equate citizens with a female CPR number as women, regardless of their gender. We are aware that a minor group (< 0.01%) of the target population includes transgender women who have undergone legal gender change.

2 Digital Post is a national mandatory cross-governmental digital service infrastructure that enables public authorities to communicate securely and digitally with Danish citizens [9].

3 We define early postmenopause (1–2 years) as equivalent to the early postmenopause stage + 1b, late early postmenopause (> 2–8 years) corresponds to the early postmenopause stage + 1c, and late postmenopause (> 8 years) is equal to the postmenopause stage + 2 in STRAW + 10 [1].

## Declarations

### Lawful basis of research

This study was conducted in the public interest for scientific research purposes. The legal basis for processing the collected data was Article 6(1)(e) and Article 9(2)(j) of the General Data Protection Regulation (GDPR), along with section 10 of the Data Protection Act, which permitted the processing of personal data exclusively for statistical and scientific purposes. All participants were informed and provided with detailed information about the study and the data processing procedures, in accordance with the obligation to inform.
