# Supplementary material for: Women in Healthy Transition (KISO) Survey: a cohort of 153,800 women aged 45–59 years living in Denmark
Source: Eur J Epidemiol. 2025 Aug 27;40(10):1251–61. doi: 10.1007/s10654-025-01291-0 (PMC12660343; doi:10.1007/s10654-025-01291-0)
Supplement: Supplementary file 1 — Supplementary file1 (PDF 308 kb) [file 10654_2025_1291_MOESM1_ESM.pdf]

Supplementary material 1
Original article
Cohort Profile: Women in Healthy Transition (KISO) Survey: A cohort of 153,800 women aged 45-59 years living in Denmark
European Journal of Epidemiology
Sigrid Normann Biener, Terese Sara Høj Jørgensen, Maria Hybholt
sibi@nexs.ku.dk

Supplementary material 1

Sensitivity analyses

Fig. S1a Stages of menopause at age 45 to 60 years with hormonal contraception users in separate group

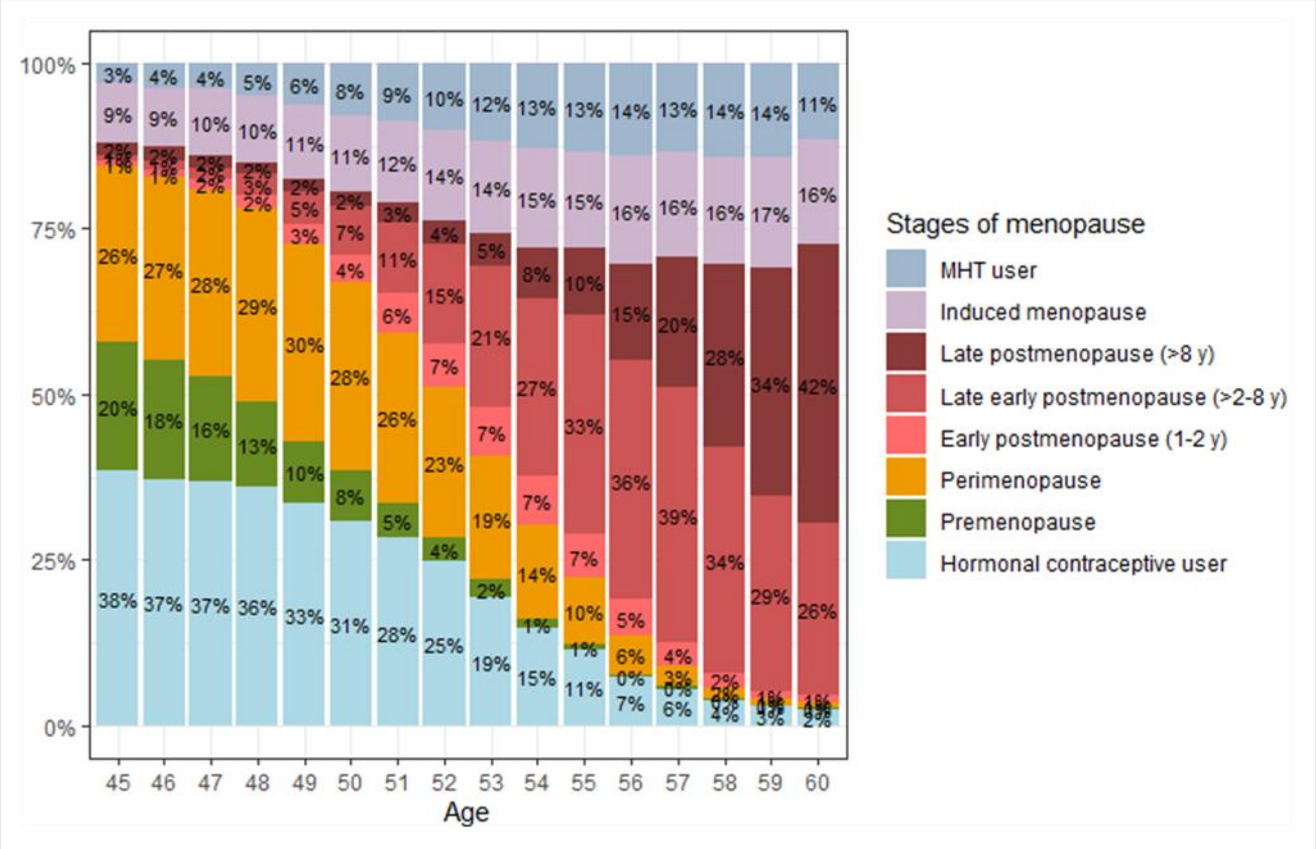

Sample size: n=152,697
Note: The age range includes up to 60 years because a minor proportion of respondents answered the questionnaire after turning 60.
Abbreviations: MHT, menopausal hormone therapy; y, years.

Fig. S1b Prevalence and severity of symptoms on stages of menopause with hormonal contraceptive users in separate group

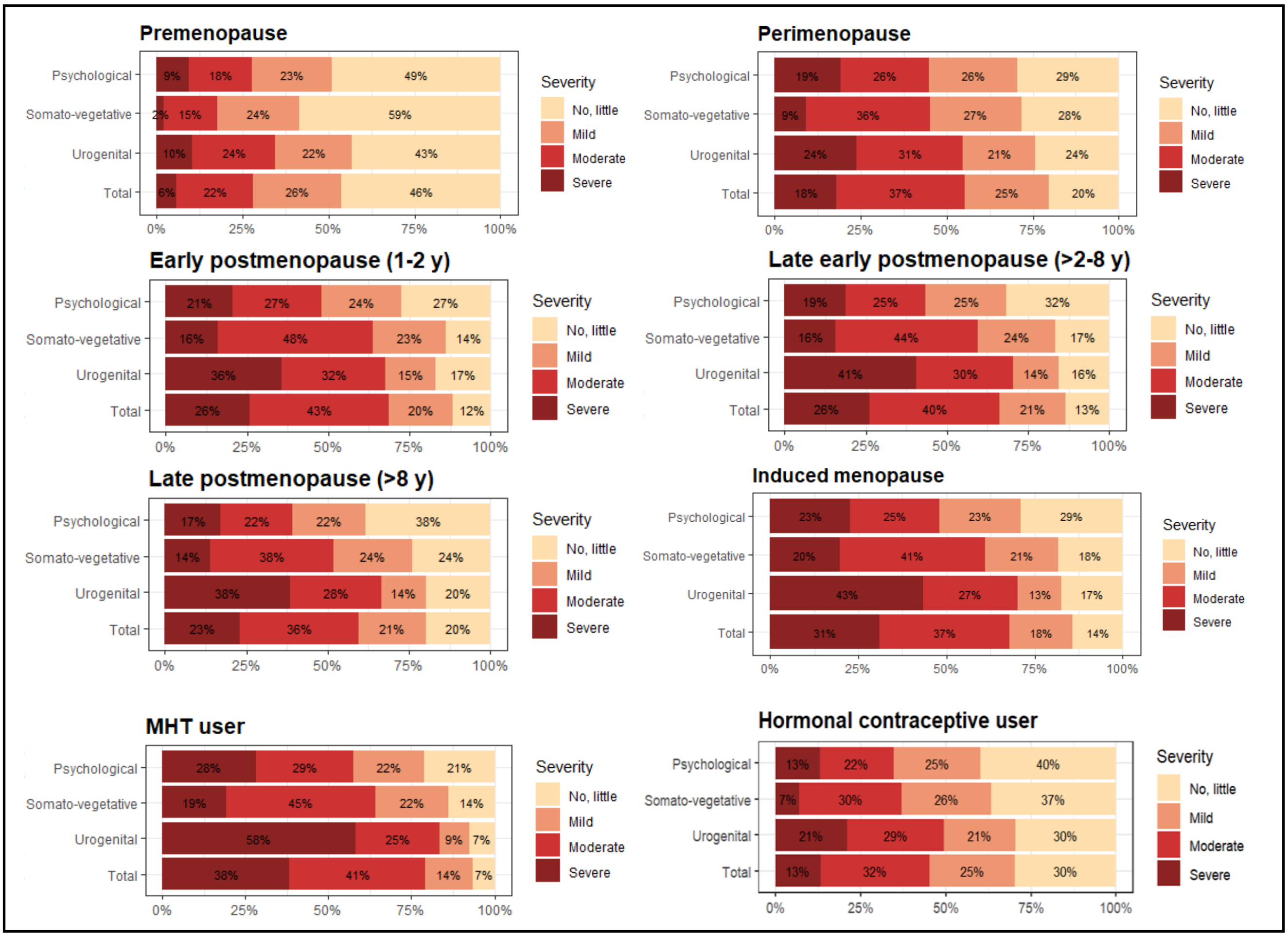

Sample size: premenopause (n=8,502), perimenopause (n=26,189*), early postmenopause (n=6,253), late early postmenopause (n=28,792*), late postmenopause (n=15,238*), induced menopause (n=20,102*), MHT user (n=15,062*), Hormonal contraceptive user (n=31,183*)

*n varies by ≤4 across the 4 sub-analyses due to missing values.

Note: Based on MRS 11-item symptoms. "Severe" and "extremely severe" were combined into "severe".

Abbreviations: MHT, menopausal hormone therapy; MRS, Menopause Rating Scale; y, years.
