# Supplementary material for: Women in Healthy Transition (KISO) Survey: a cohort of 153,800 women aged 45–59 years living in Denmark
Source: Eur J Epidemiol. 2025 Aug 27;40(10):1251–61. doi: 10.1007/s10654-025-01291-0 (PMC12660343; doi:10.1007/s10654-025-01291-0)
Supplement: Supplementary file 2 — Supplementary file2 (PDF 355 kb) [file 10654_2025_1291_MOESM2_ESM.pdf]

Supplementary material 2
Original article
Cohort Profile: Women in Healthy Transition (KISO) Survey: A cohort of 153,800 women aged 45-59 years living in Denmark
European Journal of Epidemiology
Sigrid Normann Biener, Terese Sara Høj Jørgensen, Maria Hybholt
sibi@nexs.ku.dk

Supplementary material 2

Supplementary analyses

Fig. S2a Prevalence and severity of individual symptoms in premenopause

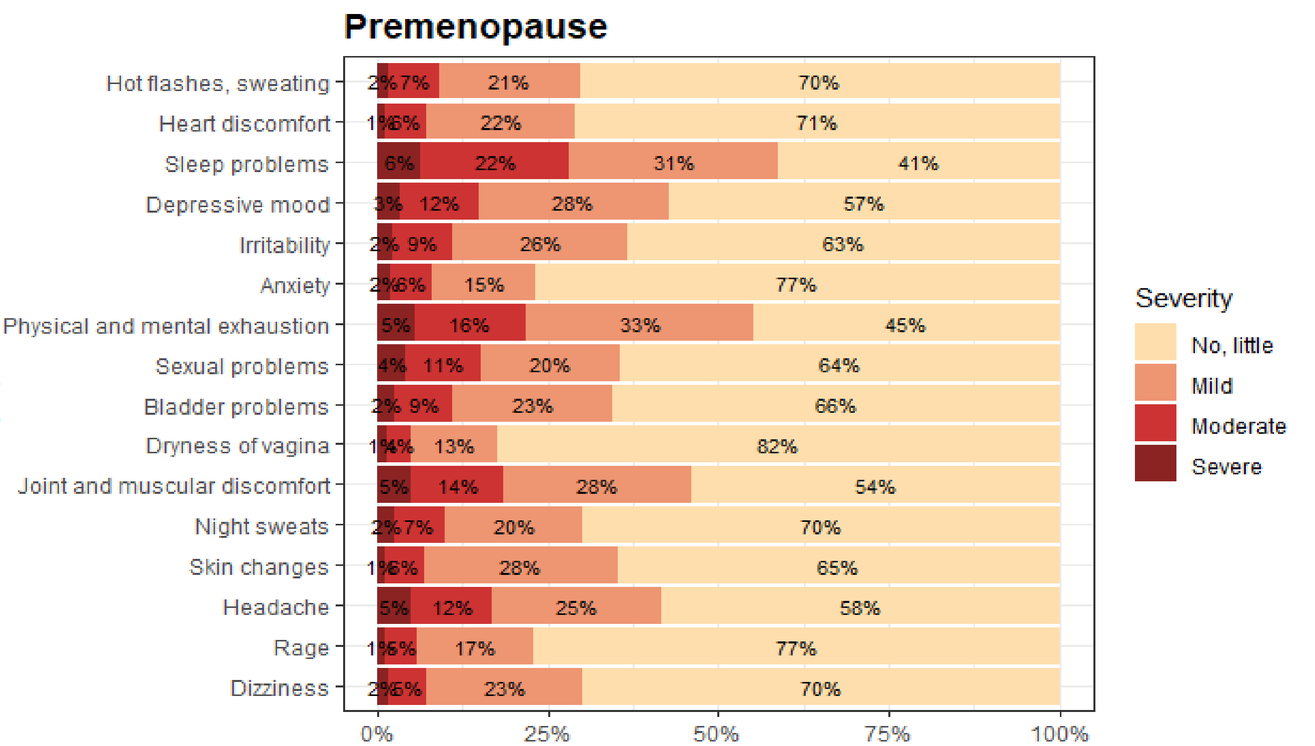

Sample size: n=11,659 (n varies by 5 across the 16 sub-analyses due to missing values).
Note: MRS 11-item symptoms and 5 additional symptoms. "Severe" and "extremely severe" were combined into "severe".
Abbreviations: MRS, Menopause Rating Scale.

Fig. S2b Prevalence and severity of individual symptoms in perimenopause

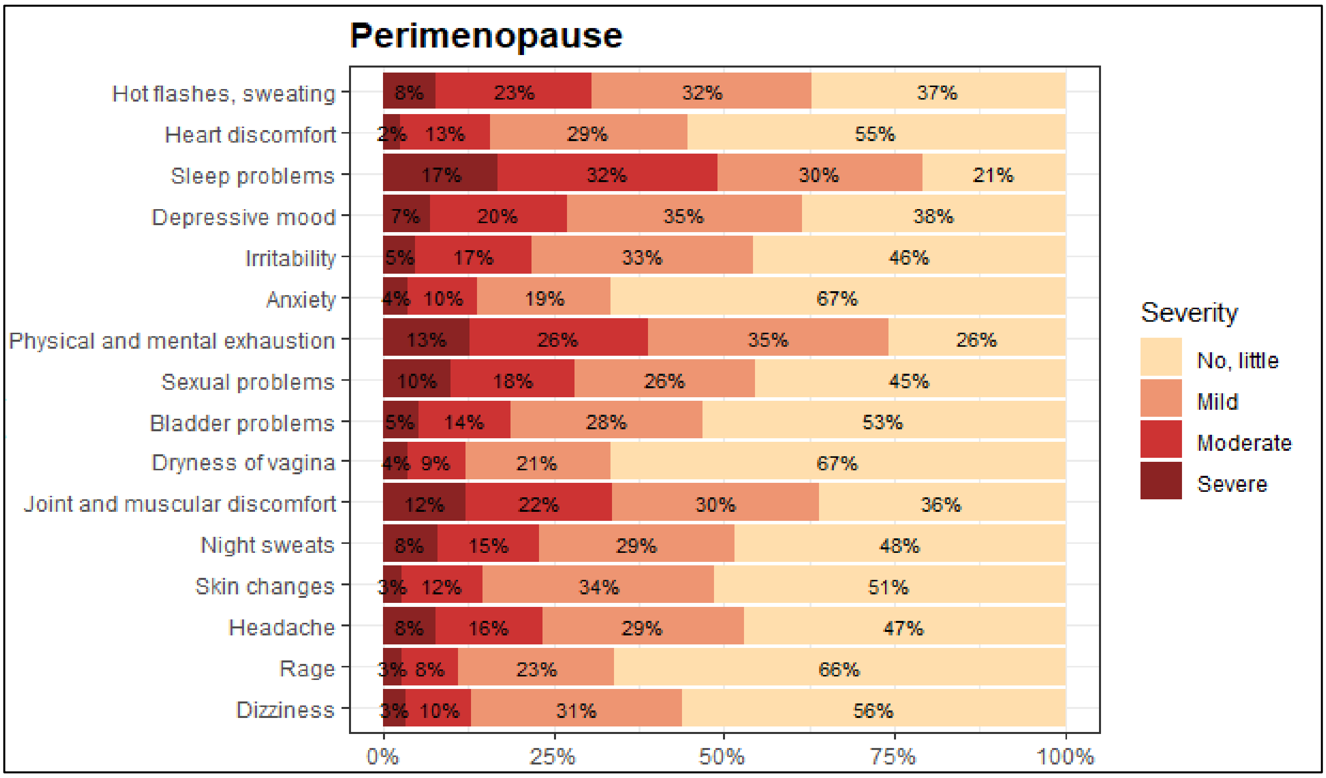

Sample size: n=36,124 (n varies by 3 across the 16 sub-analyses due to missing values).
Note: MRS 11-item symptoms and 5 additional symptoms. "Severe" and "extremely severe" were combined into "severe".
Abbreviations: MRS, Menopause Rating Scale.

Fig. S2c Prevalence and severity of individual symptoms in early postmenopause (1-2 y)

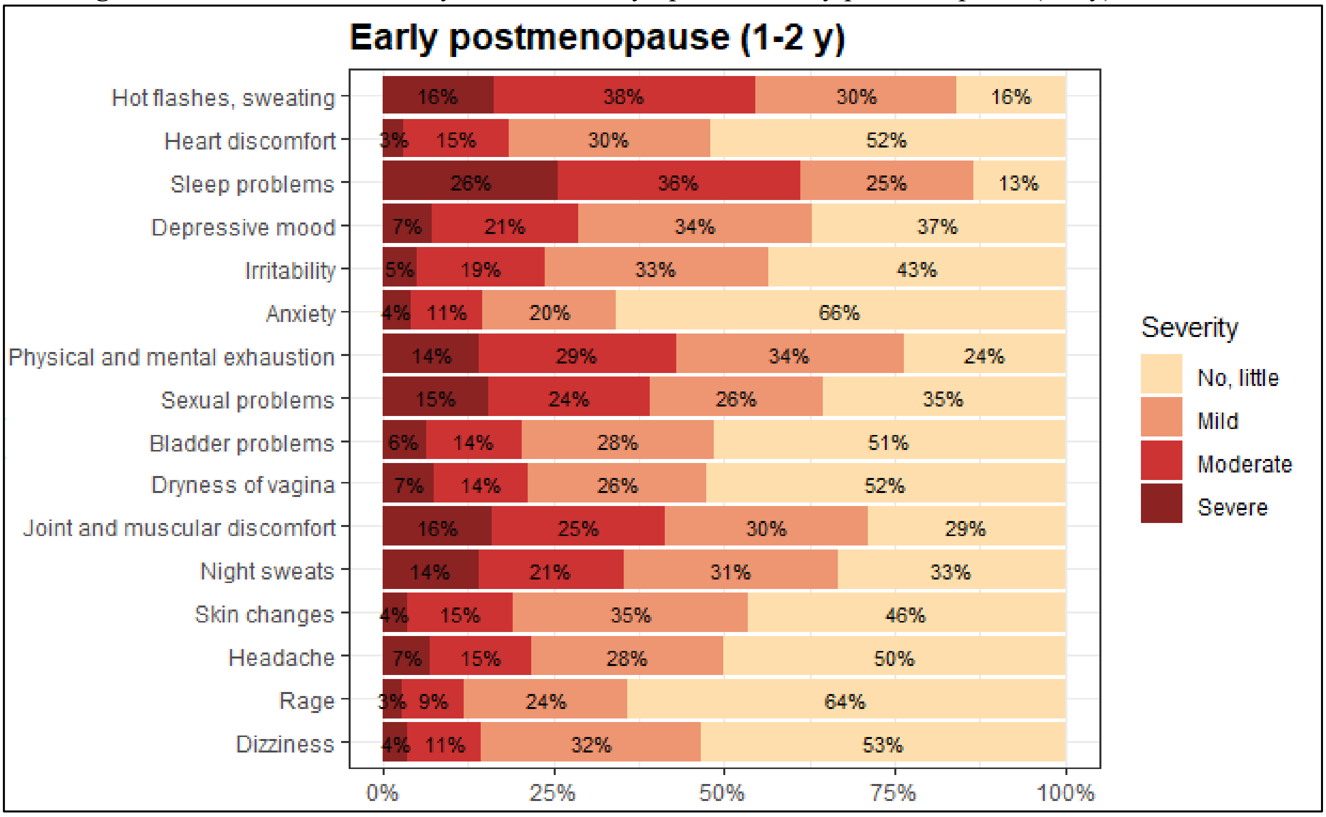

Sample size: n=8,314 (n varies by 2 across the 16 sub-analyses due to missing values).
Note: MRS 11-item symptoms and 5 additional symptoms. "Severe" and "extremely severe" were combined into "severe".
Abbreviations: MRS, Menopause Rating Scale; y, years.

Fig. S2d Prevalence and severity of individual symptoms in early postmenopause (>2-8 y)

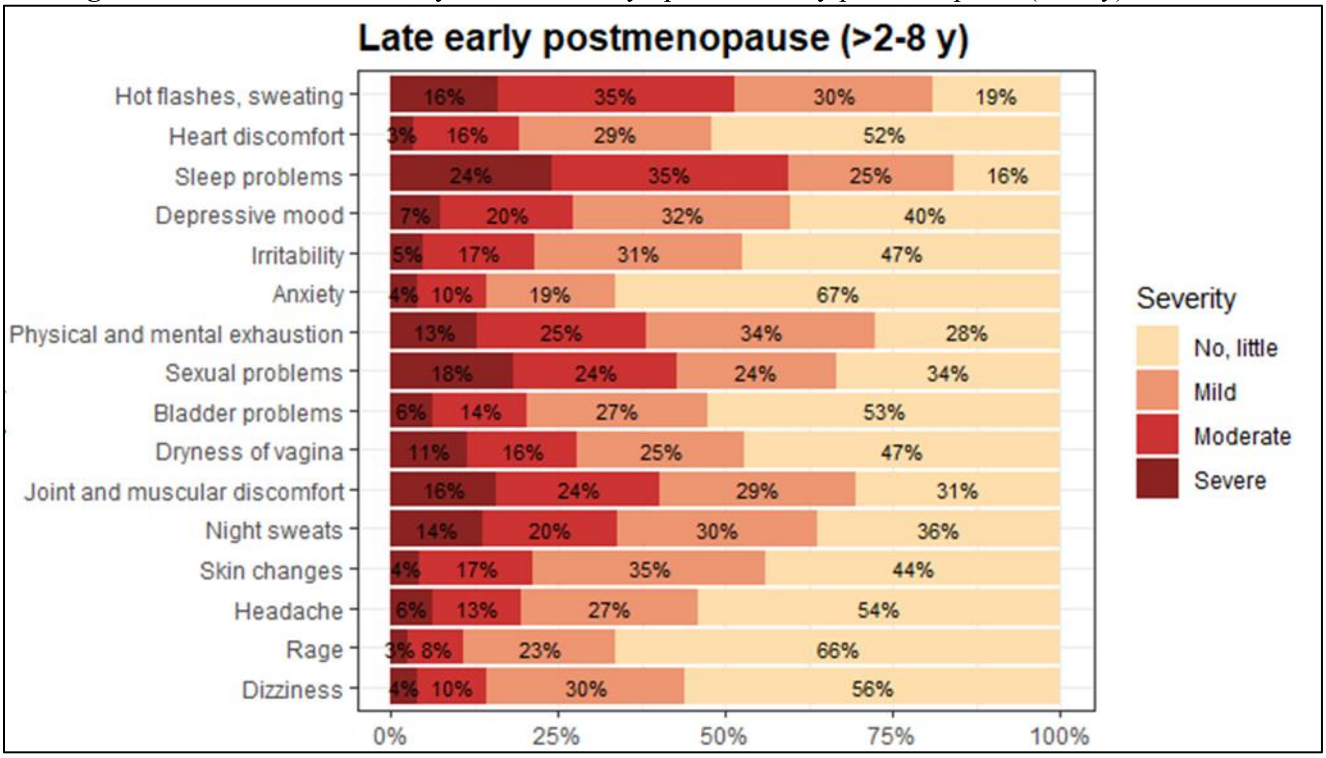

Sample size: n=36,258 (n varies by 5 across the 16 sub-analyses due to missing values).
Note: MRS 11-item symptoms and 5 additional symptoms. “Severe” and “extremely severe” were combined into “severe”.
Abbreviations: MRS, Menopause Rating Scale; y, years.

Fig. S2e Prevalence and severity of individual symptoms in early postmenopause (>8 y)

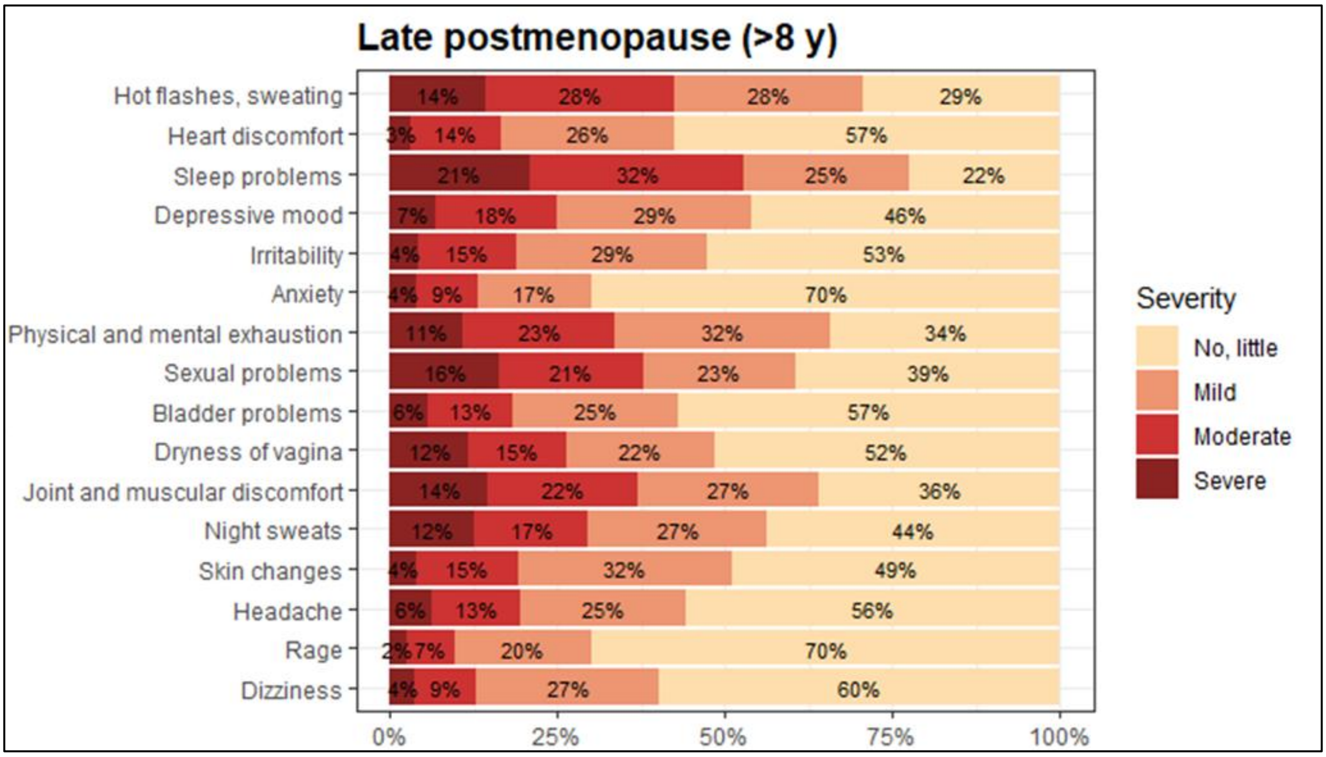

Sample size: n=21,924 (n varies by 7 across the 16 sub-analyses due to missing values).
Note: MRS 11-item symptoms and 5 additional symptoms. “Severe” and “extremely severe” were combined into “severe”.
Abbreviations: MRS, Menopause Rating Scale; y, years.

Fig. S2f Prevalence and severity of individual symptoms in induced menopause

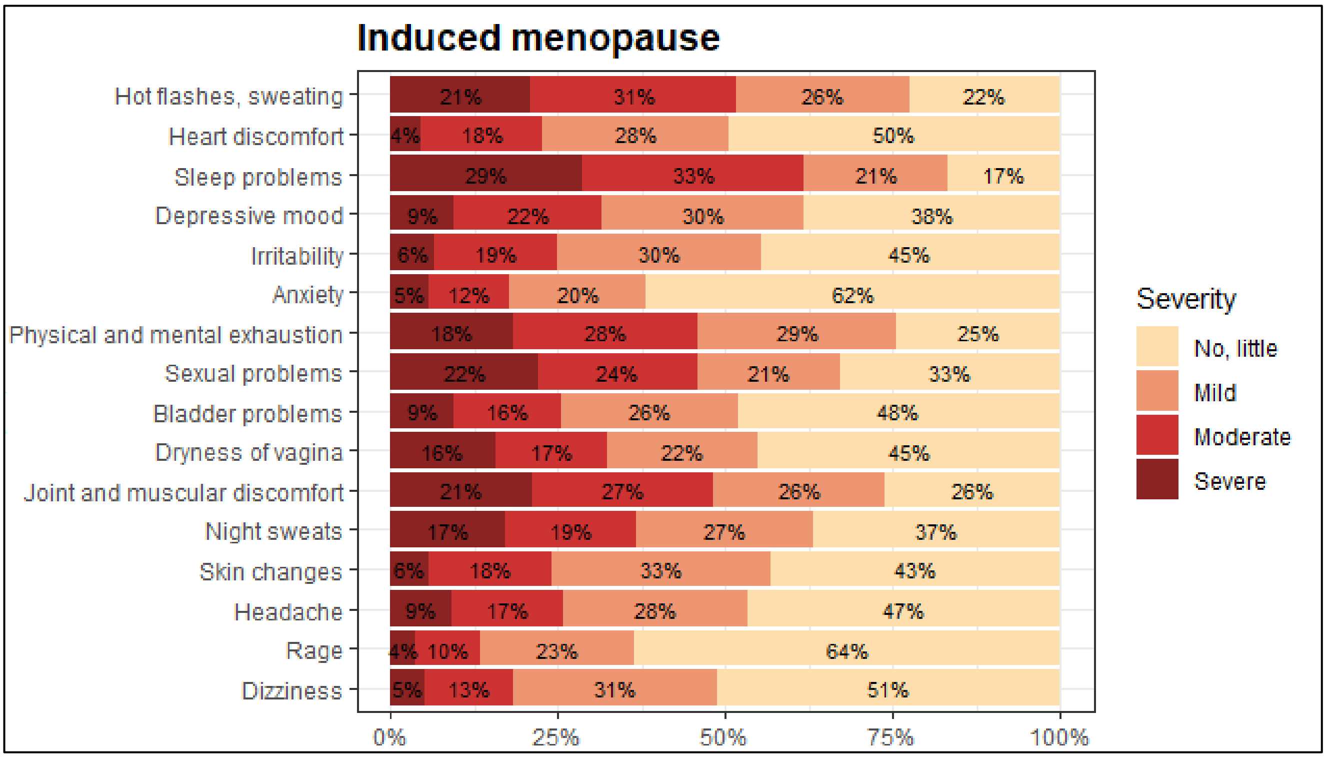

Sample size: n=20,102 (n varies by 3 across the 16 sub-analyses due to missing values).
Note: MRS 11-item symptoms and 5 additional symptoms. "Severe" and "extremely severe" were combined into "severe".
Abbreviations: MRS, Menopause Rating Scale.

Fig. S2g Prevalence and severity of individual symptoms in MHT users

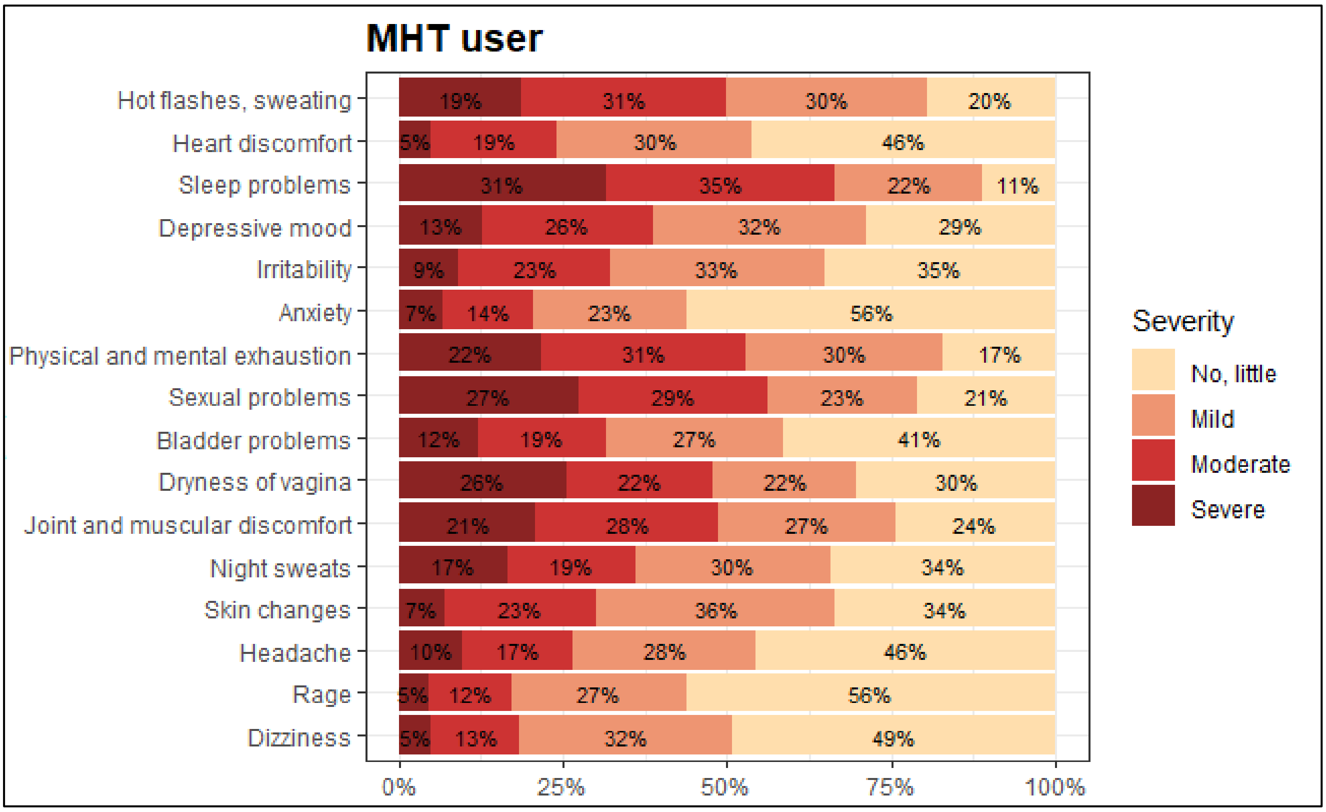

Sample size: n=15,062 (n varies by 2 across the 16 sub-analyses due to missing values).
Note: MRS 11-item symptoms and 5 additional symptoms. "Severe" and "extremely severe" were combined into "severe".
Abbreviations: MHT, menopausal hormone therapy; MRS, Menopause Rating Scale.
